# Development and Implementation of an Internal Quality Control Sample to Standardize Oligomer-Based Diagnostics of Alzheimer’s Disease

**DOI:** 10.3390/diagnostics13101702

**Published:** 2023-05-11

**Authors:** Marlene Pils, Alexandra Dybala, Fabian Rehn, Lara Blömeke, Tuyen Bujnicki, Victoria Kraemer-Schulien, Wolfgang Hoyer, Detlev Riesner, Dieter Willbold, Oliver Bannach

**Affiliations:** 1 attyloid GmbH, Merowingerplatz 1a, 40225 Düsseldorf, Germany; m.pils@attyloid.com (M.P.); alexandra.ziemski@hhu.de (A.D.); f.rehn@attyloid.com (F.R.); l.bloemeke@attyloid.com (L.B.); detlev.riesner@hhu.de (D.R.); d.willbold@fz-juelich.de (D.W.); 2 Institute of Biological Information Processing (Structural Biochemistry: IBI-7), Forschungszentrum Jülich, 52428 Jülich, Germany; t.bujnicki@fz-juelich.de (T.B.); v.kraemer-schulien@fz-juelich.de (V.K.-S.); wolfgang.hoyer@hhu.de (W.H.); 3 Institut für Physikalische Biologie, Heinrich-Heine-Universität Düsseldorf, 40225 Düsseldorf, Germany

**Keywords:** Alzheimer’s disease, diagnosis, dementia, biomarkers, amyloid-β peptide, oligomer-based diagnostics, immunoassays, internal quality control, atomic force microscopy, Shewhart chart

## Abstract

Protein misfolding and aggregation are pathological hallmarks of various neurodegenerative diseases. In Alzheimer’s disease (AD), soluble and toxic amyloid-β (Aβ) oligomers are biomarker candidates for diagnostics and drug development. However, accurate quantification of Aβ oligomers in bodily fluids is challenging because extreme sensitivity and specificity are required. We previously introduced surface-based fluorescence intensity distribution analysis (sFIDA) with single-particle sensitivity. In this report, a preparation protocol for a synthetic Aβ oligomer sample was developed. This sample was used for internal quality control (IQC) to improve standardization, quality assurance, and routine application of oligomer-based diagnostic methods. We established an aggregation protocol for Aβ1–42, characterized the oligomers by atomic force microscopy (AFM), and assessed their application in sFIDA. Globular-shaped oligomers with a median size of 2.67 nm were detected by AFM, and sFIDA analysis of the Aβ1–42 oligomers yielded a femtomolar detection limit with high assay selectivity and dilution linearity over 5 log units. Lastly, we implemented a Shewhart chart for monitoring IQC performance over time, which is another important step toward quality assurance of oligomer-based diagnostic methods.

## 1. Introduction

Alzheimer’s disease (AD) is a progressive brain disease that causes increasing deterioration of mental abilities. AD is mainly characterized by misfolding and aggregation of amyloid-β (Aβ) peptides and Tau proteins into amyloid plaques and neurofibrillary tangles [1,2]. For decades, these deposits were considered the primary cause of disease onset and progression. However, it is increasingly recognized that the soluble oligomeric species formed during the aggregation process are the major neurotoxic species of AD [3,4,5,6]. Consequently, these oligomers represent a primary drug target and a promising biomarker candidate for early AD diagnostics. The minute amounts of oligomeric Aβ in body fluids such as cerebrospinal fluid (CSF, aM-fM) [5] and excessive concentrations of Aβ monomers and matrix components require extremely sensitive and specific quantitation technologies [5,6,7].

**Figure 1:**
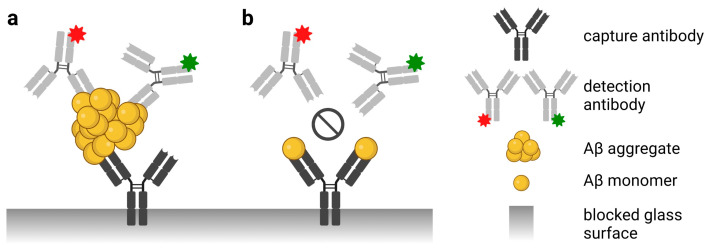
Scheme of the sFIDA principle. The biochemical principle of sFIDA is similar to a sandwich ELISA with capture and detection antibodies directed against the same or overlapping epitopes of the N-terminus of Aβ. Monomeric and oligomeric Aβ species of the sample bind to the capture antibodies. (**a**) However, the red or green fluorescently labeled detection antibodies only detect aggregated Aβ species such as oligomers because the assay antibodies bind to the same or overlapping epitope. (**b**) Therefore, the red or green labeled detection antibody cannot bind monomers because the capture antibody already masks the epitope. Subsequently, the assay surface is imaged using dual-color fluorescence microscopy (excitation at 635 and 488 nm), and only colocalized pixels above a defined cutoff threshold are counted by image data analysis. Abbreviations: Aβ, amyloid-β; sFIDA, surface-based fluorescence intensity distribution analysis. Created with BioRender.com (accessed on 26 April 2023).

We previously developed the surface-based fluorescence intensity distribution analysis (sFIDA) technology as an oligomer-specific quantitation method with single-particle sensitivity [8,9,10,11,12]. Although the biochemical setup of the sFIDA assay is similar to sandwich ELISA (Figure 1), the readout is microscopy-based with sub-femtomolar sensitivity [7]. In sFIDA, Aβ species are captured on a glass surface by an N-terminal anti-Aβ antibody; subsequently, Aβ oligomers are detected by two different fluorescence-labeled antibodies. Monomeric Aβ is not detected because capture and detection antibodies compete for the same or overlapping epitopes [11]. Using linear epitopes, all subtypes of aggregated protein, including low- and high-molecular-weight oligomers, are detected [8]. The glass surface is imaged by dual-color total internal reflection fluorescence microscopy (TIRFM) to count the number of oligomers in a sample. Background noise is reduced by applying a cutoff, which is a predefined intensity value, and only signals above the cutoff are evaluated. Moreover, signal colocalization of both fluorescently labeled detection antibodies (called sFIDA readout) increases specificity and directly correlates with the Aβ oligomer concentration in the sample [11].

Moreover, oligomer quantification is challenging because reliable calibration standards are required [4,5,13]. To address this issue, we recently developed and characterized stable silica nanoparticles (SiNaPs) coated with Aβ peptides, which serve as calibration standards for translating pixel-based readouts into molar particle concentrations [13]. In contrast to natural oligomers that can undergo structural changes and epitope masking in response to changes in buffer conditions or matrix effects [14,15], SiNaPs are very robust because of their artificial, silica-based nature. Nevertheless, it is necessary to have an Aβ oligomer-based internal quality control (IQC) sample that sensitively detects unfavorable or declining assay performance in response to analytical, biological, or clinical changes [16,17]. In addition, monitoring the day-to-day (between-run) precision and accuracy of the IQC sample improves assay comparability and standardizes oligomer-based diagnostic methods for routine applications [18].

Soluble Aβ oligomers are transient and very heterogeneous in size and shape. Thus, investigators have struggled to prepare a reliable Aβ oligomer sample for in vitro and in vivo studies [4,5,13,19]. Firstly, Aβ oligomers must be sufficiently stable during the assay procedure, i.e., must not dissociate to monomers or grow further into insoluble structures such as fibrils. In this context, the ease of the aggregation protocol would also be important, as it should not require complex pretreatments for stabilization, such as crosslinking or protein engineering [20]. Secondly, a suitable characterization method must be applied to investigate the shape and size of Aβ oligomers. Several methods are described in the literature, whereby atomic force microscopy (AFM) enables size and shape characterization because of its insensitivity to buffer components and matrix effects while providing high-resolution three-dimensional morphological images [21]. Thirdly, Aβ oligomers should be stable for in vitro studies such as oligomer-based diagnostic methods to facilitate reproducibility that is evaluated using quality assurance tools such as control sheets, Shewhart charts, or Cusum charts [16,17,18].

In the present study, we describe detailed methods to consistently generate an Aβ1–42 oligomer-based IQC sample and characterize its shape and size using atomic force microscopy. We then demonstrate IQC applicability and monitoring using sFIDA for in vitro oligomer-based diagnostic methods. We investigated several validation parameters, such as detection and quantification limits, intra-assay variability, dilution linearity, and assay selectivity. Lastly, we demonstrate the use of Shewhart charts for monitoring the IQC performance over time at three IQC sample concentrations.

## 2. Materials and Methods

### 2.1. Monomerization Aβ1–42 Peptide Stock and Aggregation Protocol

The monomerization and aggregation protocol used to generate oligomeric Aβ species was established by considering the findings of Aβ aggregation studies [19,20,21,22,23]. For a graphical illustration of the preparation methods, including the monomerization and aggregation procedure, see Figure S1.

Monomerization of the Aβ1–42 peptide stock is essential for generating homogeneous oligomers [19,21]. Thus, we used a strong fluorinated alcohol, 1,1,1,3,3,3-hexafluoro-2-propanol (HFIP, Sigma-Aldrich, St. Louis, MO, USA), to remove any preexisting β-sheet secondary structure or seeds. All preparation steps that use HFIP should be performed under a fume hood or clean bench because of its volatility. We reduced the lyophilized starting material per aliquot because Aβ1–42 aggregates more spontaneously at a stock concentration higher than 90 nM. Thus, we first prepared aliquots containing 50 µg of Aβ1–42 by adding 550 µL of HFIP directly into the original vial containing 1 mg of Aβ1–42 (Bachem AG, Bubendorf, Switzerland). Complete dissolution of the Aβ1–42 peptide was achieved by 30 min incubation at room temperature (RT) and agitated at 650 rpm. The solution was then transferred into a protein low-binding reaction tube (Eppendorf, Hamburg, Germany), and the original vial was rinsed with 550 µL of HFIP. Next, this solution was quickly divided into 20 aliquots of 50 µL each using a repeating pipette or cooled Hamilton syringe (Figure S1, step 1). Subsequently, tubes were transferred into a SpeedVac and dried for ~1 h without heating until all HFIP and H_2_O were removed. The monomeric stock tubes containing 50 µg of Aβ1–42 were sealed and stored at RT (Figure S1, step 2). To further reduce the initial amount of Aβ1–42, we repeated this step by adding 550 µL of HFIP to a 50 µg Aβ1–42 tube and creating 10 new aliquots of 5 µg each. These were dried and stored as described above (Figure S1, step 3).

For the preparation of the IQC sample stock solution, 5 µg of monomeric Aβ1–42 was solved in 5 µL of dimethyl sulfoxide (DMSO, Sigma-Aldrich), briefly mixed, spun down, and agitated for 10 min at 650 rpm (Thermomixer, Eppendorf) at RT (Figure S1, step 4). This step should be scheduled immediately prior to further use because a prolonged residence time of Aβ in the DMSO stock solution can lead to spontaneous protofibril formation [21]. Because physiological conditions such as salt concentration and pH facilitate the aggregation of oligomeric Aβ species [21], we subsequently diluted the DMSO stock solution with 1× phosphate-buffered saline, pH 7.4 (PBS, Sigma-Aldrich) containing 0.04% sodium azide (NaN_3_, AppliChem, Darmstadt, Germany) to a concentration of 10 µM (Figure S1, step 5). The IQC sample stock solution was again briefly mixed, spun down, and agitated for 16 h at 650 rpm at RT to promote oligomerization (Figure S1, step 6).

### 2.2. Atomic Force Microscopy

AFM is insensitive to matrix effects and solution conditions, and it generates detailed surface information at a nanometer scale [21]. Therefore, AFM was used for the size and shape characterization of the synthesized Aβ oligomers.

The 10 µM IQC sample stock solution containing oligomeric species was diluted to 1 µM in PBS. As a control, 10 µM monomeric IQC sample stock solution was prepared analogously, but incubation after adding PBS was not performed (Section 2.1). Ten microliters of each sample was loaded onto a mica slide and incubated for 30 min at RT in a closed petri dish. A wet tissue was added to prevent drying artefacts. The slide was washed 3× with 100 µL of ddH_2_O and dried with N_2_ gas. The samples were measured using NanoWizard III (JPK BioAFM, Bruker Corporation, Billerica, MA, USA) with an OMCL-AC160TS cantilever (Olympus Corporation, Tokyo, Japan) in the intermittent contact mode (AC mode) in air. For size determination, three images (2 × 2 µm with a resolution of 512 × 512 pixels) were recorded with a frequency of 0.5 Hz. Assuming the oligomers are globular, the height profile of 1300 oligomers was further analyzed with ImageJ using the “Find Maxima” tool. The determined height was equated to the size of the oligomers.

### 2.3. sFIDA

#### 2.3.1. Synthesis of SiNaPs Coated with Aβ1–15

In this study, SiNaPs coated with Aβ1–15 were synthesized, functionalized, and activated, as described previously [7,8,13]. Briefly, SiNaPs were synthesized via the Stöber process and silanized with 3-aminopropyl(triethoxysilane) (APTES, Sigma-Aldrich) to functionalize the surface with primary amino groups. The carboxy groups of maleimidohexanoic acid (MIHA, abcr GmbH, Karlsruhe, Germany) were then activated by 1-ethyl-3-(3-dimethylaminopropyl) carbodiimide (EDC, Sigma-Aldrich) and N-hydroxysuccinimide (NHS, Sigma-Aldrich) and coupled covalently to the amines. Aβ and maleimide groups were crosslinked using C-terminal functionalized Aβ1–15 peptides with cysteamine (Peptides and Elephants, Henningsdorf, Germany). Lastly, the molar SiNaP concentration was calculated on the basis of the silicon concentration, which was determined by inductively coupled plasma mass spectrometry, and the size, density, and shape of the particles were determined by transmission electron microscopy.

#### 2.3.2. Labeling of Antibodies

The anti-Aβ antibody IC16 (mouse, monoclonal, amino acids (aa)2–8, Heinrich-Heine-University Düsseldorf, Düsseldorf, Germany) labeled with CF633 dye (Biotium, Freemont, CA, USA) and the anti-Aβ antibody Nab228 (mouse, monoclonal, aa1–11, Sigma-Aldrich) labeled with CF488A dye (Biotium) were used as detection probes for TIRFM. Labeling was performed according to the manufacturer’s protocol. After purification via size exclusion using a polyacrylamide bead suspension (Bio-Gel P-30 Gel, Bio-Rad Laboratories, Hercules, CA, USA), the concentration and degree of labeling of both detection probes were calculated according to the manufacturer’s protocols.

#### 2.3.3. Assay Protocol

In the present study, 384-well plates (Sensoplate Plus, Greiner Bio-One, Frickenhausen, Germany) were functionalized with the Nab228 antibody (2.5 µg/mL in 0.1 M carbonate solution pH 8.4, 40 µL per well). After overnight incubation at 4 °C, plates were washed five times with Tris-buffered saline containing Tween (TBST, 1× TBS (Serva, Duisburg, Germany) and 0.05% Tween-20 (AppliChem)) and five times with 1× TBS and blocked with 0.5% bovine serum albumin (BSA, AppliChem) in 1× TBS containing 0.03% ProClin (Sigma-Aldrich,) for 1.5 h at RT. After washing the wells, as described above, 20 µL per well of SiNaPs (3.16-fold dilution, 10.26 pM–0.3 fM), assay controls, or IQC samples were applied in fourfold determination and incubated for 2 h at RT. For sample dilution, 1× PBS containing 0.05% Tween, 0.5% BSA, and 0.095% NaN_3_ was used. Using the oligomeric IQC sample stock solution, a 3.16-fold dilution series ranging from 100 nM (IQC-15) to 0.01 pM (IQC-1) was used for validation studies, whereas, for the QC chart, 20 replicates of 316 pM (IQC-10), 31.6 pM (IQC-8), and 3.16 pM (IQC-6) were used. Wells were then washed five times with 1× TBS before adding 20 µL per well of the centrifuged (100,000× *g*, 4 °C, 1 h) detection antibodies mixture (IC16-CF633 + Nab228-CF488A, each at 0.625 µg/mL, in TBST + 0.1% BSA) for 1 h at RT. Lastly, wells were washed again, and the buffer was changed to 1× TBS with 0.03% ProClin. A microplate washer (405LS Microplate Washer, BioTek, Winnoski, VT, USA) was used for all washing steps.

#### 2.3.4. Image Data Acquisition

The well surface was imaged in two different channels (channel 633: excitation 635 nm, emission filter 705/72 nm, exposure time 1000 ms, gain 1000; channel 488: excitation 488 nm, emission filter 525/36 nm, exposure time 1000 ms, gain 500) using TIRFM (Leica DMI6000B, Wetzlar, Germany). Twenty-five images per well with 1000 × 1000 pixels each were measured, representing 3.14% of the well surface.

### 2.4. Statistics

General statistical analyses were performed using Microsoft Excel (Microsoft, Redmond, WA, USA), and OriginPro (OriginLab Corporation, Northampton, MA, USA) and matlab2019b (The MathWorks, Natick, MA, USA) were used for calculations and graphs. Data were further analyzed for normal distributions using the Shapiro–Wilk test; for non-normal distributions, a non-parametric test was used, i.e., the Mann–Whitney U test.

#### 2.4.1. Analysis of Image Data

Images were analyzed using in-house developed software that features artefact filtering and an automated sFIDA readout calculation [7,8]. To reduce background noise, intensity cutoffs were defined as the signal intensity exceeding 0.05% of the total pixels of the individual channels of the used blank control (dilution buffer, BC). Lastly, the number of pixels that had intensities above the defined cutoff and were colocalized in both fluorescence channels was calculated as the sFIDA readout. sFIDAta calculated the sFIDA readout on the basis of the mean value, standard deviation, and coefficient of variation (CV%) for each sample and the respective replicates.

#### 2.4.2. Calibration

For calibrating the received sFIDA readouts, a weighted linear regression analysis with respective weights calculated as one per readout was performed with matlab2019b (The MathWorks). Therefore, all data points of the SiNaP calibration curve that differed significantly from BC and were above the limit of detection (Section 2.4.1) were included. For all further analyses, only the calibrated sFIDA readouts were shown.

#### 2.4.3. Analytical Validation: Detection and Quantification Limits

For the calculation of the limit of blank (LoB) and limit of detection (LoD), 24 BC samples were analyzed, and parameters were calculated according to Armbruster et al. [24] using Equations (1) and (2). Afterward, values were translated into particle concentrations using the calculated calibration curve.

(1) $$LoB={mean\;sFIDA\;reaodut}_{BC}+1.645\times {standard\;deviation}_{BC}.$$

(2) $$LoD=mean\;{sFIDA\;readout}_{BC}+2\times {standard\;deviation}_{BC}.$$

Using the calibrated particle concentrations of the Aβ oligomer dilution series, the linear working range was defined by calculating the lower and the upper endpoint and the dilution linearity. Therefore, the concentrations that differed significantly from the next lower concentration were determined using the one-sided Mann–Whitney U test with a confidence interval of 5%. Before calculating dilution linearity, background correction was performed by subtracting the BC value from each IQC sample value. Subsequently, the percentage dilution linearity of each dilution step was calculated using Equation (3).

(3) $$Dilution\;linearity\left[\%\right]=\frac{observed\;value}{\left(expected\;value/dilution\;factor\right)}\times 100\%$$

Within the working range, the mean dilution linearity should be 80–120%, and the coefficient of determination should be higher than 0.95 to be accepted. The CV% of the four replicates of the same sample within the same run was calculated to assess intra-assay variability (within-run precision).

#### 2.4.4. Analytical Validation: Analytical Selectivity

The selectivity of sFIDA indicated by the percentage signal reduction (Equation (4)) was carried out by measuring the IQC-13 (10 nM monomer concentration) sample in different assay setups. Nonspecific binding to the blocking agent used was excluded by performing capture control, where the capture antibody was omitted. As an autofluorescence control, the assay was performed using only TBST + 0.1% BSA without detection probes. In addition, the cross-reactivity of anti-Tau antibodies against the produced Aβ oligomers was tested. To this end, the Tau12 antibody (mouse, monoclonal, aa6–18, Biolegend, San Diego, CA, USA) was conjugated with CF633 and CF488A according to the protocol in Section 2.3.2 and diluted in TBST + 0.1% BSA to 0.625 µg/mL. The insensitivity of sFIDA against monomeric Aβ species was evaluated by applying 10 nM of freshly diluted monomeric Aβ1–42. IQC-13 was spiked in bovine CSF to simulate matrix effects, and the sFIDA readouts, generated using 0.05% cutoff-based CSF-blank, were compared to an equal concentration in the dilution buffer.

(4) $$Signal\;reduction\left[\%\right]=\left(1-\frac{observed\;readout\;assay\;control}{readout\;reference}\right)\times 100\%$$

Furthermore, sFIDA readouts of the respective assay control were compared to the readouts of the standard assay setup using the one-sided Mann–Whitney U test with a confidence interval of 5%.

#### 2.4.5. Establishment of QC-Tool

A Shewhart chart, the most widely used tool for IQC [18], was used to monitor the readouts of IQC-10, IQC-8, and IQC-6. The oligomeric IQC sample stock solution (Section 2.1) was serially diluted 20 times to corresponding monomer concentrations of 316 pM (IQC-10), 31.6 pM (IQC-8), and 3.16 pM (IQC-6) to simulate 20 observations of each IQC sample. Subsequently, each dilution underwent a fourfold sFIDA analysis, and the observed sFIDA readouts were calibrated into particle concentrations. Lastly, observed particle concentrations were plotted as absolute values against the number of analyses. Using the mean and the standard deviation of the 20 observations, the lower and upper control limits (LCL/UCL) and action limits (LAL/UAL), respectively, were calculated according to Equations (5)–(8) and were integrated into the control chart.

(5) LCL=mean−2×standard deviation

(6) UCL=mean+2×standard deviation

(7) LAL=mean−3×standard deviation

(8) UAL=mean+3×standard deviation

In general, values within the control limits are considered satisfactory. Even if values are located between control and action limits, they are still accepted if they do not affect more than 10% of the measured values. However, if one result of a sample occurs outside the action limit, or if nine consecutive results create a trend (decreasing or increasing) or lie on one side of the central line, the operator’s intervention becomes necessary [18,25]. Between-run precision was considered satisfactory when all results were unbiased, all results lay within the warning limits, and the mean CV% of the 20 replicates was below 20%.

## 3. Results

The aim of this study was to develop and characterize an IQC sample for oligomer-based diagnostic assays. The first part of the results describes the characterization of the IQC sample, which was prepared using the protocol established in Section 2.1. In the second part, we used the IQC sample in the sFIDA assay and determined several validation parameters. Lastly, application of the Shewhart chart to monitor IQC performance was demonstrated with three IQC samples.

### 3.1. Aβ Oligomer-Based IQC Sample Displays High Homogeneity

**Figure 2:**
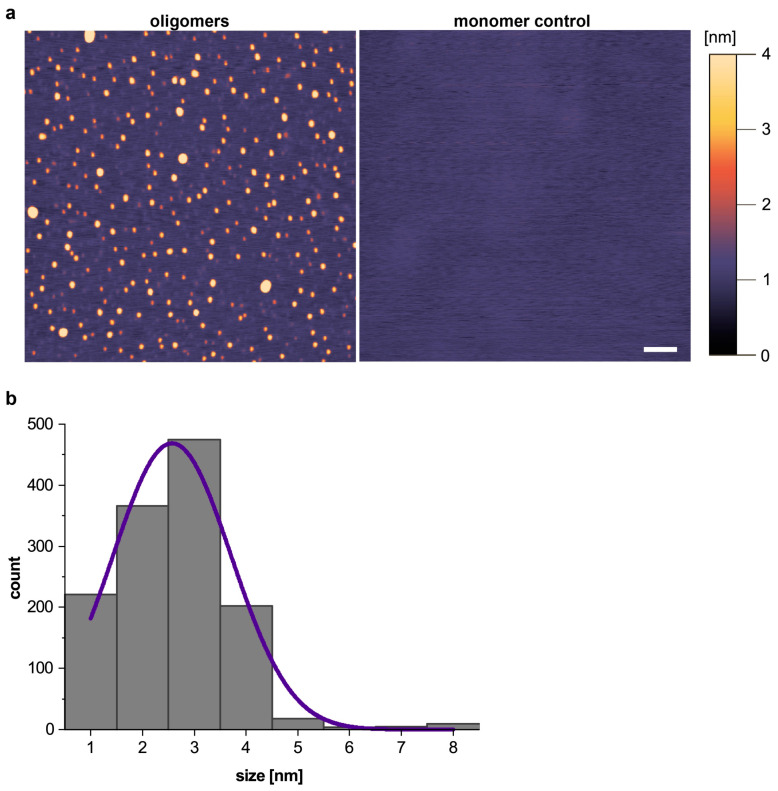
Analysis of the Aβ oligomers measured by AFM. (**a**) AFM images of the monomer control and Aβ oligomer samples. The color scale indicates the height profile. Scale bar = 200 nm. (**b**) Histogram showing the size distribution of 1300 oligomers. Abbreviations: Aβ, amyloid-β; AFM, atomic force microscopy.

The size distribution and morphology of the Aβ oligomers were determined by AFM. The analysis showed that the oligomers were monodisperse and globular in shape (Figure 2a). In contrast, we observed no particles in the monomer control containing a 10-fold higher protein concentration. Size distribution analysis revealed that the median height of all 1300 oligomers was 2.67 nm, with a minimum size of 1.07 nm. Only 2% of the detected oligomers were ≥5 nm (Figure 2b).

### 3.2. Successful Application of the Aβ Oligomer-Based IQC Sample in the sFIDA Assay

The applicability of the prepared Aβ oligomers as an IQC sample for in vitro oligomer-based diagnostic methods using the sFIDA assay was investigated. We prepared a 3.16-fold concentration series of the oligomers in dilution buffer and subjected each sample to sFIDA analysis in quadruplicate determination. The molar concentrations represent monomer concentrations ranging from 0.01 pM (IQC-1) to 100 nM (IQC-15). Using the SiNaPs calibration curve (y = 5.08x − 0.25), we calculated the Aβ oligomer concentrations in each IQC sample from the sFIDA readouts. Individual sFIDA readouts and calibrated particle concentrations for each IQC sample are listed in Table S1.

**Figure 3:**
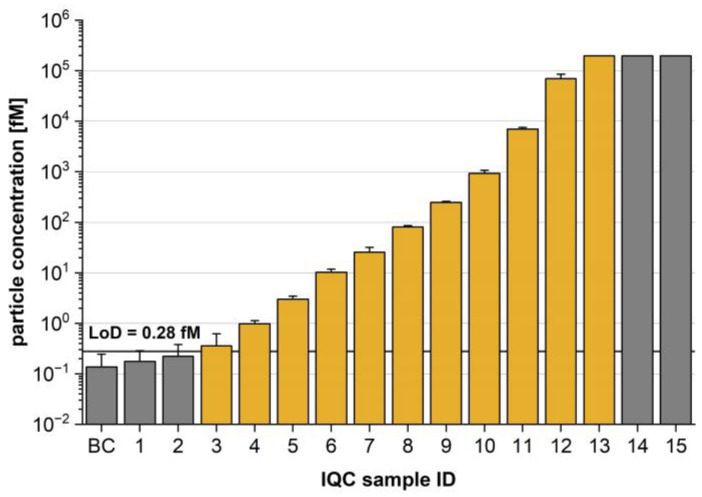
Molar particle concentration of internal quality control (IQC) samples and blank control (BC). On the basis of 24 replicates of the BC, the limit of detection (LoD) was calculated and translated into a particle concentration of 0.28 fM using the calibration curve. The linear working range (yellow) was identified by acceptable dilution linearity between the upper (196 pM) and lower (0.36 fM) limits of quantification. Note the logarithmic scale. Data are presented as the mean and standard deviation of four replicates.

Figure 3 illustrates the sFIDA IQC performance, which exhibits a 5 log dynamic range and an analytical sensitivity below the femtomolar level (LoB: 0.25 fM, LoD: 0.28 fM). The lower limit of quantification (LLoQ), upper limit of quantification (ULoQ), and acceptable dilution linearity (acceptance range of 80–120%, Section 2.4.1) defined the working range of the used IQC samples. Data were not normally distributed (*p*-value: 6.97 × 10^−6^). Thus, the two quantification limits were identified using the one-sided Mann–Whitney U test with a confidence interval of 5% (Table S1) and were set to a particle concentration of 0.36 fM (IQC-3) and 197 pM (IQC-14). Within this range, a mean percentage dilution linearity of 107% was determined (Table S2). Interestingly, a coefficient of determination of 0.73 indicated that the dilution linearity could be improved. A closer inspection of the data indicated that the IQC-14 readout was an outlier with a similar readout to that of IQC-13. Removing IQC-14 from the fit yielded a coefficient of determination of 0.99. This refit resulted in a change in the percentage dilution linearity; however, the value of 109% was still acceptable. Furthermore, within this linear working range of 0.36 fM–196 pM, a mean CV% of 18.4% was determined, indicating acceptable intra-assay variance for single-particle analysis.

### 3.3. sFIDA Features High Selectivity for the Aβ Oligomer-Based IQC Sample

**Figure 4:**
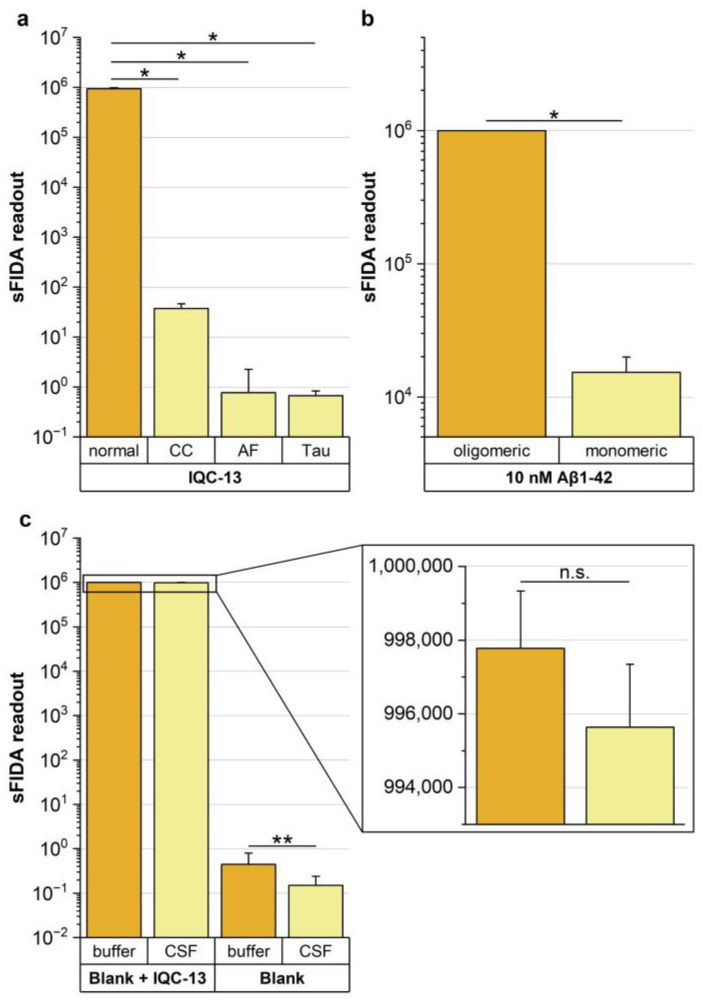
Comparison of sFIDA readout of IQC-13 applied on different assay setups. (**a**) sFIDA readouts of the normal assay setup were compared to assay setups without the capture antibody (capture control, CC), without Aβ-specific detection probes (TBST + 0.1% BSA without any detection probes, autofluorescence control, AF) or with Tau-specific detection probes (equimolar mixture of Tau12 antibodies labeled with CF633 and CF488A in TBST + 0.1% BSA, Tau). A signal reduction of almost 100% and significantly lower sFIDA readouts compared to the standard assay setup (normal, *p*-values between 0.0152 and 0.0147) were observed for all controls. (**b**) In addition, equal molar concentrations of monomeric Aβ1–42 were applied on the assay surface to demonstrate that the assay is insensitive toward monomers. A signal reduction of nearly 99% and significantly lower sFIDA readouts compared to the standard assay setup were observed (*p*-value: 0.0152), showing that interference from monomeric Aβ can be excluded. (**c**) To evaluate the matrix effects of CSF, IQC-13 was spiked into bovine CSF, and sFIDA readouts were calculated on the basis of the background signal of the additionally applied bovine CSF-blank. For IQC-13, no significant difference in the sFIDA readouts was observed (*p*-value: 0.108), and only a slight signal reduction of 0.2% was observed. In contrast, the sFIDA readout of CSF-blank was significantly lower compared to the buffer control (*p*-value: 0.002, signal reduction: 67%). Note the logarithmic scale. Data are presented as the mean and standard deviation of four replicates. Significant differences between groups were calculated using the one-sided Mann–Whitney U test with a confidence interval of 5%. Significant differences are marked as follows: * 0.01 ≤ *p* < 0.05; ** 0.001 ≤ *p* < 0.01; n.s. not significant. Abbreviations: CSF, cerebrospinal fluid; IQC, internal quality control.

Testing different assay controls confirmed that sFIDA is highly selective for aggregated Aβ species and robust against false-positive signals because of matrix interference and cross-reactivities. Figure 4a shows sFIDA readouts of IQC-13 applied on different assay setups. For all controls, a signal reduction of almost 100% was observed (Table S3a). Because data showed a non-normal distribution (Shapiro–Wilk test *p*-value: 2.53 × 10^−4^), we applied the nonparametric one-sided Mann–Whitney U test with a confidence interval of 5% to investigate differences between the sFIDA readouts of the respective assay control and the reference. For all controls, significantly lower sFIDA readouts were observed compared to the reference (for individual *p*-values, see Table S3). Aβ oligomers were only detected when captured on the assay surface via the anti-Aβ antibodies, whereas no detection was observed in the absence of the capture antibody. In addition, the capture control readout was significantly lower than the reference values (*p*-value: 0.0152). Moreover, false-positive signals generated by the autofluorescence of chemicals and buffers were not detected because the sFIDA readout of the autofluorescence control was significantly lower than the sFIDA readout of the reference, as indicated by the *p*-value of 0.0147. Using anti-Tau antibodies as the detection probe or monomers (Figure 4b, Table S3b) as the target yielded no false-positive signals caused by cross-reactivity. For both controls, significantly lower sFIDA readouts compared to the reference were observed (*p*-value of 0.0152).

Furthermore, after spiking the Aβ oligomers in CSF, only negligible matrix effects were observed, with a minor signal reduction of 0.2% and no significant difference in the readouts (*p*-value: 0.108) (Figure 4c, Table S3c). In addition, matrix components in the CSF-blank reduced the background signal more efficiently than the dilution buffer, indicated by a signal reduction of approximately 67% and significantly lower readouts indicated by a *p*-value of 0.002 (CSF-blank vs. BC). Consequently, the signal-to-noise ratio between IQC-13 and the respective blank was three times higher in CSF than in the dilution buffer.

### 3.4. Shewhart Chart as a Reliable QC-Tool for Monitoring IQC Performance

Control charts are a valuable tool for monitoring assay performance and can be used by any laboratory [25]. In this work, we demonstrated the use of a Shewhart chart to monitor the performance of three IQC samples in sFIDA (for respective TIRFM images showing co-localized pixel above cutoff values, see Figure S2) by integrating values of 20 observations of each IQC sample into a separate chart (Table S4). Charts were then interpreted analogously to defined rules (Section 2.4.3).

**Figure 5:**
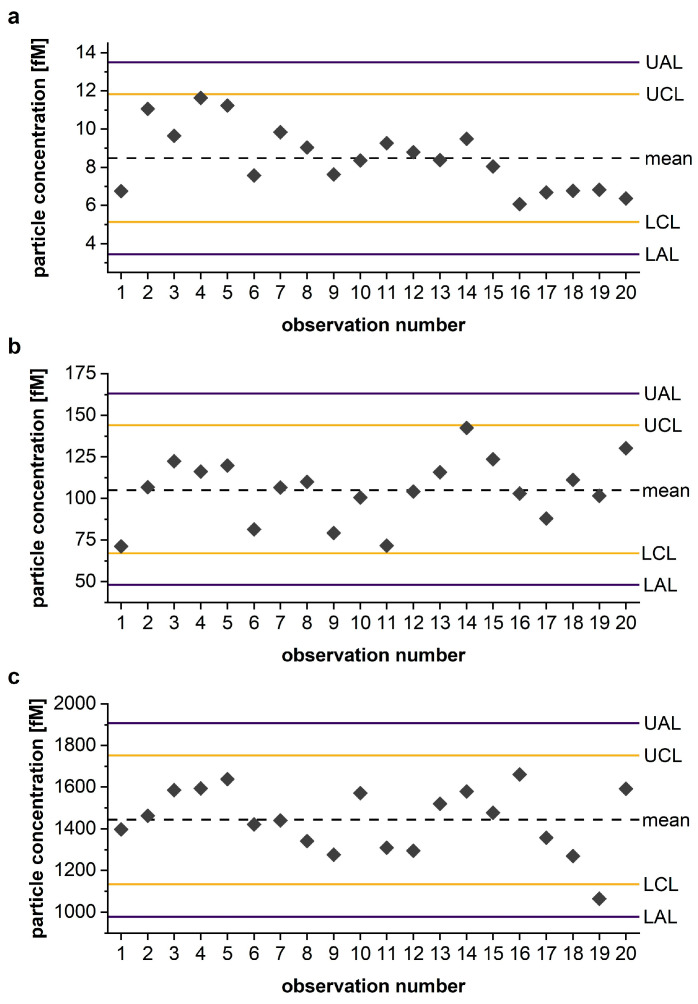
Shewhart chart monitoring the performance of IQC-6 (**a**), IQC-8 (**b**) and IQC-10 (**c**). The calibrated particle concentrations of 20 observations of each IQC sample (gray dots) were plotted to simulate assay performance in chronological order. Mean: central dashed line; upper and lower control limit (UCL, LCL): yellow line; upper and lower action limit (UAL, LAL): purple line. Data are presented as the mean of four replicates (gray data points). Abbreviation: IQC, internal quality control.

All three IQC samples showed low inter-assay variability as the calculated mean CV% of the respective 20 observations was below 20% (IQC-6 = 16.2%, IQC-8 = 16.5%, IQC-10 = 17.6%). As illustrated in Figure 5, none of the IQC samples exceeded the action limit. In addition, neither IQC-6 nor IQC-8 exceeded the control limits, but one value of sample IQC-10 occurred between the control and action limit. Nonetheless, assay performance was considered acceptable within the predefined range because this was the only deviation. No ascending or descending trend of the nine consecutive observation points was seen in IQC samples. Even if no out-of-control situation could be determined in IQC samples, IQC-6 should be monitored further as a general downward progression became apparent. Moreover, an out-of-control situation may arise in the foreseeable future if the next three observation points of IQC-6 also occur between the central line and the lower control limit.

In particular, for those assays subjected to quality management, such as sFIDA technology, troubleshooting out-of-order situations can be monitored rapidly, as possible causes related to the operator, instruments, manufacturing protocols, and/or lot numbers of assay components are regularly documented.

## 4. Discussion

In the present study, we developed a homogeneous and reproducible Aβ oligomer-based IQC sample for sFIDA and established a QC-tool for monitoring assay performance.

We characterized the formation of Aβ oligomers by AFM, which revealed globular-shaped oligomers with a median size of 2.67 nm, whereby AFM analysis revealed the size of a dried oligomer and not the hydrodynamic size. Oligomers are in thermodynamic equilibrium with monomers and larger aggregates, such as amyloid fibrils. In vitro produced oligomers have been shown to dissociate into monomers within a few hours, with only a small fraction of oligomers converting to fibrils [26]. Conversion to fibrils can be excluded herein because no fibrillary morphologies were detected by AFM. Noteworthily, the sFIDA assay currently does not discriminate oligomers from larger yet still soluble aggregates because of the diffraction limit of the optical detection system. However, as larger particles bind more fluorescent probes, a size determination of sub-resolution particles should be possible based on pixel intensity. For this purpose, the oligomers developed can serve as a size standard. In addition to established AD biomarkers, such as monomeric Aβ1–42, the ratio of Aβ1–42 to Aβ1–40, phosphorylated Tau, and total Tau in CSF, and magnetic resonance imaging and positron emission tomography (PET), i.e., Amyloid-PET or Tau-PET [2,27,28], oligomeric forms of Aβ represent a promising biomarker candidate for early AD diagnosis [5,6,29]. Consequently, in vitro, oligomer-based diagnostics are the subject of current biomarker research.

We detected and quantified oligomers at sub-femtomolar concentrations, down to 0.28 fM (LoD), which qualifies the sFIDA assay for biofluid-based in vitro oligomer-based diagnostic methods [5]. We previously showed that Aβ oligomers and aggregates are detectable in human CSF samples [10] and complex matrices such as murine and human brain homogenate samples [9] using sFIDA. Due to further development of the assay protocol, including the use of synthetic Aβ oligomers shown herein, the analytical sensitivity was further improved compared to our previously published data [7]. The potential of this analytical sensitivity to improve diagnostic performance must be validated using a large set of clinical samples. In comparison to other oligomer-specific Aβ assays, sFIDA ensures single-particle sensitivity. Although a direct comparison of sensitivity among different assays is challenging because of variations in assay design, calibration standards, and detection limit calculations, we evaluated the sensitivity ranges of various assays (Table 1).

Only the assay described by Savage et al. (0.08 fM) [30] or the commercial ELISA assay from Immuno-Biological Laboratories Co. Ltd. (IBL, Fujioka, Japan) [31] (31.4 fM), which also claims to detect single particles, offers sensitivity in the low femtomolar range, which is presumably because of the combined use of N-terminal and oligomer-specific assay antibodies. In contrast, all listed homotypic assays, using the same antibody for capture and detection, showed insufficient sensitivity levels between 91 and 720 fM. However, because of epitope competition, these assays [32,33,34] and the sFIDA assay are insensitive toward monomers.

Through our experiments, we demonstrated that Aβ oligomer-based IQC samples are reproducible, homogeneous, and suitable in oligomer-based diagnostic methods such as sFIDA. However, as our studies were performed in an artificial sample matrix, further validation data from authentic biological sample matrices such as the plasma or CSF should be obtained, including extensive recovery and parallelism studies [35]. In addition, the critical concentration at which endogenous components compromise the readout and lead to false-positive or false-negative results must be investigated. For sFIDA analysis of α-synuclein and Tau aggregates in CSF, we showed that blood contamination and HAMAs can affect the sFIDA readout [8]. In particular, for CSF, validation should be straightforward because we determined a signal loss of only 0.2% for IQC-15 due to matrix effects. The Aβ oligomer IQC can also facilitate the development of a blood-based sFIDA assay, which is currently in progress. However, the complex plasma matrix could make validation more challenging, as strong matrix effects such as interference between Aβ and human serum albumin or between circulating human antibodies with the assay antibodies are expected [6,15,36,37]. In addition to matrix effects, sample stability and sample tubing can also influence the measurement signal [38,39]. For future routine applications and regulatory approval of the sFIDA technology, the IQC sample should ideally be available as a ready-to-use kit component. Hence, the benchtop stability and long-term stability of the IQC sample should be investigated by testing different sample tubes, storage temperatures and durations, and the effects of repetitive freeze–thaw cycles [2,35,40].

## 5. Conclusions

Since protein misfolding and aggregation are pathological hallmarks for a variety of neurodegenerative diseases, it is necessary to establish additional IQC samples, e.g., based on Tau or α-synuclein oligomers that improve the routine application of oligomer-based diagnostic methods such as sFIDA. Transitioning from research use to in vitro diagnostics (IVD) has various regulatory requirements [11]. The Aβ oligomer-based IQC sample implemented represents an important step toward the standardization, routine application and ultimately, registration of sFIDA as a diagnostic tool of AD for the IVD market.

## Figures and Tables

**Table 1:** Comparison of several Aβ oligomer quantification assays according to their sensitivity level. For this purpose, the respective weight concentrations pg/mL were converted into femtomolar oligomer concentrations using the approximate molecular weights of the used calibration standards.

Reference	Assay Setup	Calibration Standard	Sensitivity [fM]
sFIDA	Single-particle analysis with fluorescence microscopy, overlapping epitopes capture: Nab228 aa1–11 detection: IC16 aa2–8 + Nab228 aa1–11	Aβ1–15 SiNaPs	LoD = 0.28
Savage et al. [30]	Single particle analysis with bead-based assay capture: 19.3 oligomer-specific detection: 82E1 aa1–16	Aβ1–42 oligomers (MW 1117 kDa)	LoD = 0.08
IBL [31]	ELISA capture: 82E1 aa1–16 detection: 24B3 oligomer-specific	E22P–Aβ40 Dimer	31.4 (N/A)
Hölttä et al. [32]	ELISA, overlapping epitopes capture and detection: 82E1 aa1–16	Dimer Aβ1–11	LLoQ = 90.9
Kasai et al. [33]	ELISA, overlapping epitopes capture and detection: Ban50 aa1–10	MAP 16-mer (lysine core)	LoD = 190
Esparza et al. [34]	Single particle analysis with bead-based assay overlapping epitopes capture and detection: HJ3.4 aa1–13	Aβ1–40Ser26Cys dimer	LLoQ = 720

## Data Availability

The data presented in this study are available in the article and its Supplementary Materials.

## Supplementary Materials

The following supporting information can be downloaded at https://www.mdpi.com/article/10.3390/diagnostics13101702/s1: Figure S1. Scheme of monomerization and oligomerization procedure; Figure S2. TIRFM-images of colocalized pixels of IQC-6, IQC-8, and IQC-10 samples with intensities above blank control-based cutoff; Table S1. Individual sFIDA readouts, calibrated molar particle concentrations, and CV% for each IQC sample; Table S2. Calculation of dilution linearity of Aβ oligomer-based IQC samples within working range; Table S3. Selectivity and recovery of the sFIDA assay to IQC-13; Table S4. Raw data used to generate Shewhart charts in Figure 5.
