# Supplementary material for: Development and Implementation of an Internal Quality Control Sample to Standardize Oligomer-Based Diagnostics of Alzheimer’s Disease
Source: Diagnostics (Basel). 2023 May 11;13(10):1702. doi: 10.3390/diagnostics13101702 (PMC10217173; doi:10.3390/diagnostics13101702)
Supplement: Supplementary file 1 [file diagnostics-13-01702-s001.zip › diagnostics-2346664-supplementary.pdf]

Development and Implementation of an Internal Quality Control Sample to Standardize Oligomer-Based Diagnostics of Alzheimer's Disease

Supplementary Material

Marlene Pils, Alexandra Dybala, Fabian Rehn, Lara Blömeke, Tuyen Bujnicki,
Victoria Kraemer-Schulien, Wolfgang Hoyer, Detlev Riesner, Dieter Willbold and Oliver Bannach

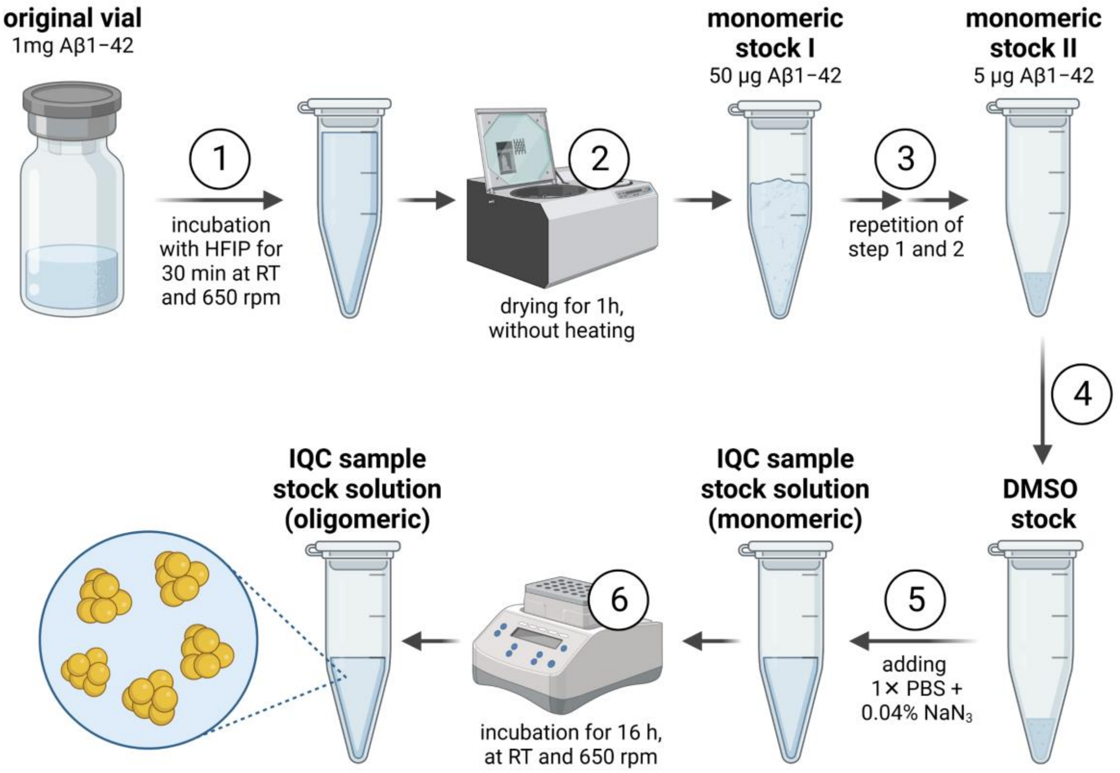

Figure S1. Scheme of monomerization and oligomerization procedure. Step 1: 1 mg Aβ1−42 is dissolved 550 µl HFIP for 1 h at RT with mixing at 650 rpm. The original sample tube is rinsed with an additional 550 µl HFIP, and the combined sample is divided into twenty aliquots containing 50 µg Aβ1−42. Step 2: aliquots are transferred to a SpeedVac and dried for ~1 h without heating until all HFIP and H_2O are removed. Step 3: the initial amount of Aβ1−42 is reduced by dissolving 50 µg Aβ1−42 with 550 µl HFIP and dividing this sample into ten aliquots containing 5 µg Aβ1−42. These aliquots are dried using a SpeedVac. Step 4: for oligomerization, 5 µg Aβ1−42 is dissolved in 5 µl DMSO, mixed briefly, centrifuged, and agitated for 10 min at RT and 650 rpm. Step 5: An IQC stock solution is prepared by adding 1× PBS containing 0.04% NaN_3. Step 6: the monomeric solution is mixed briefly, centrifuged and agitated for 16 h at 650 rpm and RT to promote oligomerization. Abbreviations: Aβ, amyloid-β; DMSO, dimethyl sulfoxide; HFIP, 1,1,1,3,3,3-hexafluoro-2-propanol; IQC, internal quality control; NaN_3, sodium azide; PBS, phosphate-buffered saline; RT, room temperature. Created with Biorender.com (accessed on 10 May 2023).

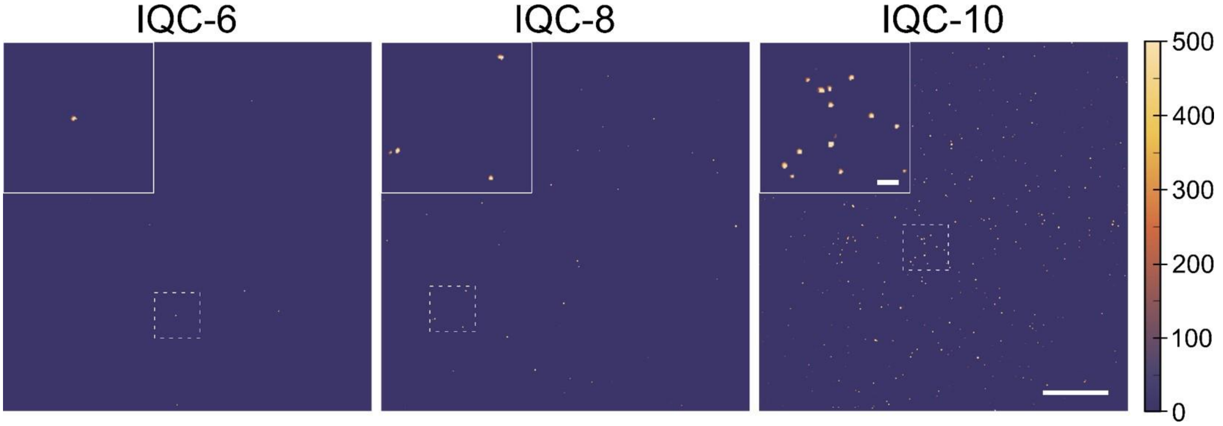

Figure S2. TIRFM-images of colocalized pixels of IQC-6, IQC-8 and IQC-10 samples with intensities above the blank control-based cutoff. The colocalized TIRFM images were created using a multi-step process. First, a global cutoff value was selected for each channel based on the blank control (0.05%). Only signals exceeding this value were considered in the consecutive analysis, enabling a distinction between signals and background noise, thereby increasing the measurement accuracy. In the next step, the matrices of both channels, representing the same area of a well, were used to create two supportive matrices. A binary matrix was generated that contains a one for all pixels whose intensity in both channel matrices is above the respective cutoff and a zero otherwise. The second matrix was the product of both channel matrices, which was scaled to the original range of values by dividing through by the maximum possible intensity value. The cutoffs were considered by multiplying the two generated supportive matrices, and the colocalization matrix for a distinct area of the well was created. For each image, a section was selected and enlarged (10×) to demonstrate differences in particle concentrations among the tested IQC samples. The color scaling was created using matlab2019b, where all pixels with an intensity equal to or higher than 500 were colored yellow. Scale bar image: 20 µm; scale bar enlarged image: 2 µm. Abbreviations: IQC, internal quality control; TIRFM, total internal reflection fluorescence microscopy.

Table S1. Individual sFIDA readouts, calibrated molar particle concentrations and CV% for each IQC sample. sFIDA readouts of each IQC sample were converted into femtomolar concentrations using the linear equation y = 5.08x – 0.25. Using the one-sided Mann-Whitney U test with a confidence interval of 5%, the molar particle concentrations of IQC samples that differed significantly from the next lower concentration were determined (marked green), and thus the lower limit of quantification (LLoQ, italic, *) and upper limit of quantification (ULoQ, italic, **) were identified. Observed sFIDA readouts are presented as the means of four replicates.

IQC sample	Monomer concentration [pM]	sFIDA readout	Particle concentration [fM]	CV%	One-sided Mann-Whitney U test *p*-value
**IQC-1**	0.01	0.7	0.18	61.3	0.1252
**IQC-2**	0.03	0.9	0.22	68.8	0.3429
**IQC-3**	0.1	1.6	0.36	73.3	*0.1714**
**IQC-4**	0.3	4.7	0.98	16.4	0.0286
**IQC-5**	1.0	15	3.0	15.7	0.0286
**IQC-6**	3.2	52	10.3	13.8	0.0143
**IQC-7**	10	130	25.7	24.1	0.0143
**IQC-8**	31.6	409	80.6	7.3	0.0143
**IQC-9**	100	1253	247	4.6	0.0143
**IQC-10**	316	4701	925	16.2	0.0143
**IQC-11**	1000	35,723	7031	7.7	0.0143
**IQC-12**	3162	351,702	69,219	23.3	0.0143
**IQC-13**	10,000	997,783	196,375	0.2	0.0143
**IQC-14**	31,622	1,000,000	196,812	0	*0.0143***
**IQC-15**	100,000	1,000,000	196,812	0	1

*LLoQ, ** ULoQ

Table S2. Calculation of the dilution linearity of Aβ oligomer-based internal quality control (IQC) samples within the working range. Data are presented as the means of four replicates. Molar particle concentrations were background corrected, and the percent dilution linearity of each IQC sample was calculated according to Equation (3). In addition, the average of all IQC samples (mean) was calculated. *IQC-14 was excluded from the analysis. Tolerance range: 80–120%.

IQC sample	Particle concentration [fM]	Particle concentration after background correction [fM]	Linearity [%]
**IQC-3**	0.36	0.22	84
**IQC-4**	0.98	0.84	93
**IQC-5**	3.0	2.9	89
**IQC-6**	10.3	10.2	126
**IQC-7**	25.7	25.5	100
**IQC-8**	80.6	80.5	103
**IQC-9**	247	247	84
**IQC-10**	925	925	42
**IQC-11**	7031	7031	32
**IQC-12**	69,219	69,219	111
**IQC-13**	196,375	196,375	316
**IQC-14**	196,812	196,812	316
		**Mean**	**107 I 109***
		**Coefficient of determination**	**0.73 I 0.99***

Table S3. Selectivity and recovery of the sFIDA assay to IQC-13. (**a**) sFIDA readouts of the IQC-13 sample were applied on different assay control setups, and the calculated percentage signal reduction was compared to the standard assay setup (normal). (**b**) Investigation of monomeric interference. (**c**) Influence of matrix effects on sFIDA readouts of the IQC-13 sample spiked in buffer or bovine CSF. Observed sFIDA readouts are presented as the means of four replicates. Using the one-sided Mann-Whitney U test with a confidence interval of 5%, the sFIDA readout of the respective assay control was compared to the readout of the standard assay setup. Abbreviations: CSF, cerebrospinal fluid; IQC, internal quality control.

(a)

**Assay control setup**	**Observed sFIDA readout**	**Signal reduction [%]**	**One-sided Mann-Whitney U test *p*-value**
**Normal**	941,271	-	-
**Capture Control (CC)**	37.27	100	0.0152
**Autofluorescence control (AF)**	0.77	100	0.0147
**Cross-reactivity anti-Tau antibodies (Tau)**	0.67	100	0.0152

(b)

**Assay control setup**	**Observed sFIDA readout**	**Signal reduction [%]**	**One-sided Mann-Whitney U test *p*-value**
**Normal**	941,271	-	-
**Monomer control**	15,231	98.38	0.0152

(c)

**Assay control setup**		**Observed sFIDA readout**	**Signal reduction [%]**	**One-sided Mann-Whitney U test *p*-value**	**Signal-to-noise ratio IQC/blank**
**Blank**	buffer	0.45	66.67	-	-
	CSF	0.15		0.002	-
**Blank + IQC-13**	buffer	997,783	0.21	-	2,217,295
	CSF	995,639		0.108	6,637,597

Table S4. Raw data used to generate Shewhart charts in Figure 5. Listed are the calibrated particle concentrations of 20 observations of each applied internal quality control (IQC) sample (IQC-6, IQC-8 and IQC-10) and the determined intra-assay variance described as CV%. The respective values were used to calculate the mean, standard deviation (SD) and upper/lower control (UCL/LCL) and action limits (UAL/LAL). Data from each observation represent the mean of four replicates.

	**IQC-6**		**IQC-8**		**IQC-10**	
**Observation**	**Particle concentration [fM]**	**CV%**	**Particle concentration [fM]**	**CV%**	**Particle concentration [fM]**	**CV%**
**1**	6.8	3.4	71.3	16.2	1397.7	24.2
**2**	11.1	18.5	106.8	19.9	1463.0	9.6
**3**	9.6	33.9	122.5	15.5	1585.6	16.5
**4**	11.6	10.2	116.3	24.4	1593.7	11.8
**5**	11.2	14.7	119.8	17.0	1638.6	20.3
**6**	7.6	36.2	81.4	20.8	1421.9	24.3
**7**	9.8	14.1	106.7	20.4	1439.7	22.6
**8**	9.0	11.0	110.1	15.3	1340.8	9.5
**9**	7.6	15.7	79.3	17.8	1276.4	20.6
**10**	8.4	22.9	100.7	27.6	1571.9	16.4
**11**	9.3	14.4	71.7	4.3	1309.4	11.8
**12**	8.8	29.6	104.3	25.1	1295.2	5.6
**13**	8.4	8.5	115.8	8.5	1520.7	32.0
**14**	9.5	8.4	142.3	7.9	1579.4	21.0
**15**	8.0	9.9	123.6	13.8	1476.9	21.9
**16**	6.1	8.7	103.1	18.8	1661.7	14.8
**17**	6.7	12.2	88.1	12.8	1356.5	12.6
**18**	6.8	19.5	111.2	14.2	1269.8	16.2
**19**	6.8	20.2	101.6	18.3	1064.7	10.8
**20**	6.4	12.1	130.3	10.6	1592.7	28.8
**Mean**	**8.5**		**105**		**1443**	
**SD**	**1.7**		**19**		**155**	
**UCL**	**11.8**		**144**		**1752**	
**LCL**	**5.1**		**67**		**1133**	
**UAL**	**13.5**		**163**		**1907**	
**LAL**	**3.5**		**48**		**978**	
